# Gadoxetic Acid-Enhanced Hepatobiliary-Phase Magnetic Resonance Imaging for Pyrrolizidine Alkaloid-Induced Hepatic Sinusoidal Obstruction Syndrome and Association with Liver Function

**DOI:** 10.1038/s41598-018-37775-1

**Published:** 2019-02-04

**Authors:** Tingting Guo, Xin Li, Xiaoqian Yang, Xiangquan Kong, Hui Liu, Tao Bai, Keshu Xu, Jin Ye, Yuhu Song

**Affiliations:** 1 Department of Radiology, Union Hospital, Tongji Medical College, Huazhong University of Science and Technology, Wuhan, 430022 China; 2 Division of Gastroenterology, Union Hospital, Tongji Medical College, Huazhong University of Science and Technology, Wuhan, 430022 China; 3 12Sigma Technologies, Shanghai, 200000 China

## Abstract

Hepatic sinusoidal obstruction syndrome (HSOS) can be caused by pyrrolizidine alkaloids(PAs)-containing herbals. In this study, the aim of our study was to investigate the imaging features of PAs-induced HSOS on gadoxetic acid-enhanced magnetic resonance imaging (MRI), susceptibility-weighted imaging(SWI) and T2* weighted imaging (T2* WI). We analyzed medical records and MR images of 28 PAs-induced HSOS patients enrolled from Feb, 2013, to Apr, 2017. Abnormal liver function was observed in most of the PAs-induced HSOS patients. Heterogeneity of liver parenchyma in hepatobillary phase (HBP) of gadoxetic acid-enhanced MR scan was observed in 100% of the PAs-induced HSOS patients. Distributional patterns of heterogeneous hypointensity were multifocal distribution (mild) in 4 patients (14.29%), multifocal distribution (severe) in 15 cases (53.57%), and diffuse distribution in 9 patients (32.14%). Hypointense in SWI and T2*WI was observed in the patients of PAs-induced HSOS, and the distribution of hypointense in SWI and T2*WI was similar to that of portal-venous phase of MR scan. The severity of heterogeneous hypointensity scored by volume fraction in hepatobillary phase of gadoxetic acid-enhanced MRI was positively correlated with PT and INR, the severity of hypointensity in HBP was a risk factor of death events. In conclusion: Heterogenous hypointensity of liver parenchyma was an imaging sign of hepatobillary phase in gadoxetic acid-enhanced MRI; thus, it will provide evidences for the diagnosis of PA-induced HSOS.

## Introduction

The intake of pyrrolizidine alkaloids (PAs)-containing products is a major etiology of hepatic sinusoidal obstruction syndrome (HSOS) in China^1–5^. To date, more than 6,000 plant species containing PAs have been identified^2^. Gynura segetum is one of the most frequent herbal medicine containing PAs, which has been used as a remedy for bone fractures, joint inflammation, pain relief, improvement of circulation, and wound healing in China^3,6^. Pyrrolizidine alkaloids exhibits hepatotoxic activity through toxic destruction of hepatic sinusoidal endothelial cells^7,8^. The diagnosis of PAs-induced HSOS usually depends on a history of taking herbs that contain PAs, and a classical triad of weight gain, painful hepatomegaly, and jaundice^3,5,9–11^. In clinical practice, PAs exposure is always obscure owing to variability of the plant components, mislabeling or misidentification of the plant, and outright contaminations. Thus, a suitable strategy should be developed to provide an assistance in diagnosing PAs-induced HSOS.

Imaging modalities have been widely used in the diagnosis of hepatic vascular diseases. Recently, several studies have described the imaging features of contrast-enhanced CT in the patients with PAs-induced HSOS, patchy liver enhancement and heterogeneous hypoattenuation were valuable features of CT scan^9,11–14^. In addition, we and Zhou H *et al*. described the imaging findings of dynamic contrast-enhanced (DCE) magnetic resonance imaging (MRI) in the PAs-induced HSOS patients. However, previous study was limited to dynamic contrast-enhanced (DCE) MRI^9,10^. Recently, Gadolinium ethoxybenzyl diethylenetriamine pentaacetic acid (Gd-EOB-DPTA),or gadoxetic acid (Primovist, Bayer Schering Pharma, Berlin, Germany) for short, a liver-specific MRI contrast agent has been increasingly used in the diagnosis of focal liver lesions^15,16^. Gadoxetic acid-enhanced MRI provides not only information about the vascular phases, but also additional information regarding the hepatobiliary phase (HBP). Recent studies demonstrated gadoxetic acid-enhanced MR imaging was useful to detect oxaliplatin-induced HSOS in the patients with metastatic colon cancer^17,18^. To our knowledge, the imaging features of gadoxetic acid-enhanced MRI have not been previously reported in the PAs-induced HSOS patients. Thus, the aim of this study was to retrospectively describe the imaging signs of gadoxetic acid-enhanced MRI in the PAs-induced HSOS patients, and then to determine the correlation between imaging features and a panel of liver function tests.

## Results

### The characteristics of enrolled patients with PAs-induced HSOS

Table 1 illustrated the relevant demographic and clinical information of PAs-induced HSOS at the time of baseline evaluation. As shown Table 1, older age and male gender seemed to be more frequently affected because the intake of PAs-containing plants occurred frequently to older age and male gender. The values of erythrocyte and leukocyte were in normal range in most of the PAs-induced HSOS patients; while, the value of platelet was abnormal in 35.71% of the patients. Then, clinical chemistry tests demonstrated that abnormal liver function was observed in most of PAs-induced HSOS patients, which was revealed by a panel of liver function tests including ALT, AST, ALP, γ-GT, total bilirubin, albumin, and PT (Table 1).

**Table 1:** Laboratory tests of the patients during the initial examination.

Patients with available data	
Age, years (mean ± SD)	61.46 ± 6.09
Gender, *n* (M/F)	M20/F8
**Peripheral blood routine examination**	
Erythrocytes, 10^12^/L (Mean ± SD)	4.64 ± 0.51
Leukocyte, 10^9^/L (Mean ± SD)	6.13 ± 1.79
PLT, ×10^9^/L(Mean ± SD)	120.14 ± 67.20
**Biochemistry**	
ALT, U/L (mean ± SD)	97.96 ± 85.41
AST	116.11 ± 97.55
ALP, U/L (mean ± SD)	179.68 ± 112.06
γ-GT, U/L (mean ± SD)	163.64 ± 112.52
Total bilirubin, μmol/L (mean ± SD)	59.00 ± 56.28
Albumin, g/L (mean ± SD)	31.67 ± 4.82
PT	17.04 ± 2.59

Note: ALT, alanine aminotransferase; AST, Aspartate aminotransferase; ALP, alkaline phosphatase; γ-GT, γ-glutamyl transpeptidase; TB, total bilirubin; PT, prothrombin time.

### The findings of gadoxetic acid-enhanced, susceptibility-weighted and T2* weighted MR imaging in PAs-induced HSOS patients

**Figure 1:**
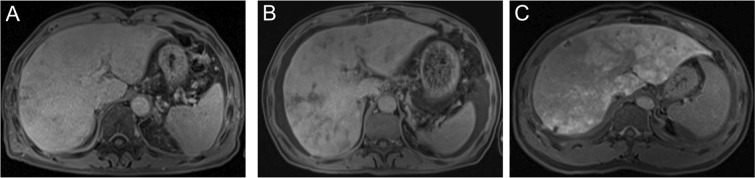
The distribution of hypointensity in hepatobiliary phase of gadoxetic acid-enhanced MRI. (**A**) Multifocal distribution (mild); (**B**) multifocal distribution (severe); (**C**) diffuse distribution.

**Figure 2:**
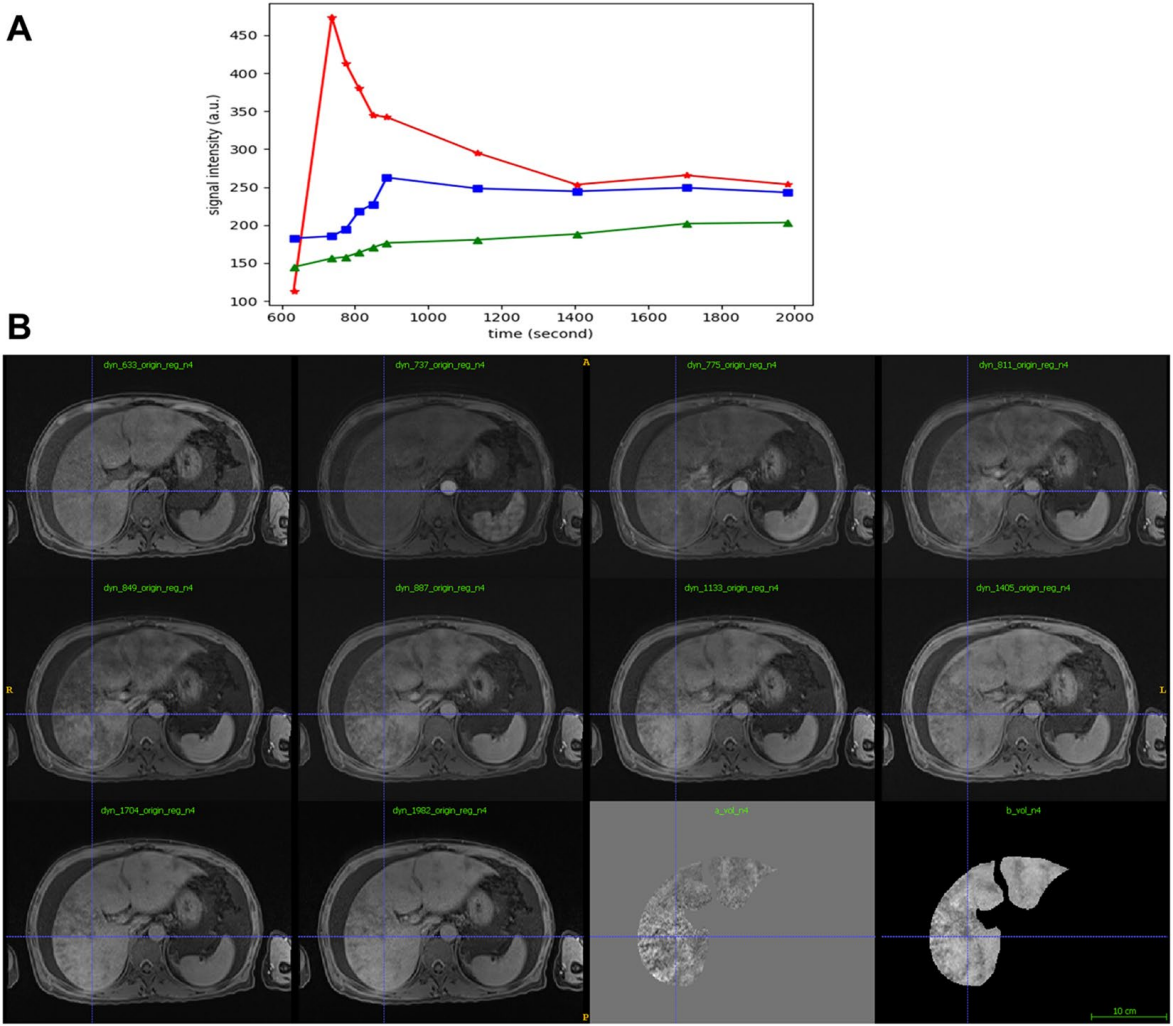
Schematic diagrams of the strategy for calculating the severity of hypointensity in hepatobiliary phase (HBP) of gadoxetic acid-enhanced MRI using volume fraction. (**A**) Plots shows its intensity curve for artery (red), normal parenchyma (blue) and unnormal parenchyma (green). (**B**) Images: pixelwise linear fitting of intensity for hepatic biliary phases, where volume ‘a’ represents the slope volume, and volume ‘b’ represents the offset volume. The unnormal region is classified as hypointensity in offset volume and hyperintensity in slop volume.

**Figure 3:**
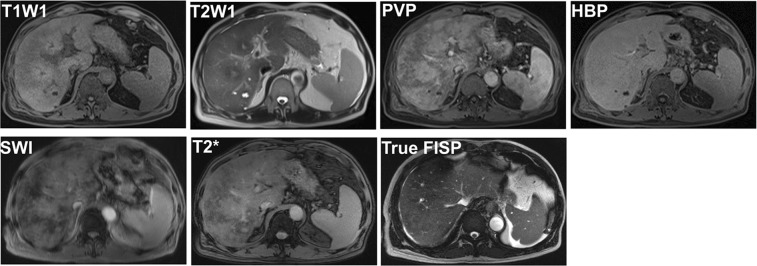
67-year-old man with PAs-induced HSOS received MRI scan, and the images of MRI SWI and T2*WI displayed the lesion more clearly than pre-contrast sequences (T1W1 and T2W1). Similar distribution of hypointensity was observed in SWI/T2*WI and portal-venous phase (PVP) of dynamic contrast-enhanced (DCE) MRI; while, distributional similarity between SWI/T2* imaging and HBP was not observed. PVP: portal-venous phase; HBP: hepatobiliary phase.

**Figure 4:**
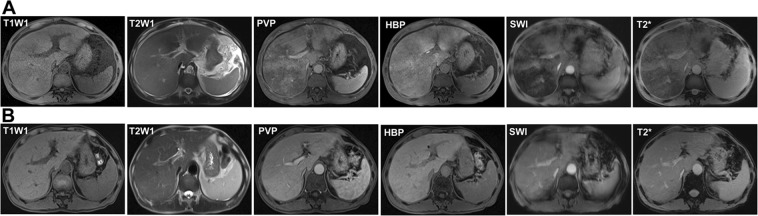
59-year-old man was diagnosed with PAs-induced HSOS after the ingestion of gynura segetum, and he received gadoxetic acid-enhanced MRI scan at the baseline visit and follow-up visit. (**A**) The images of gadoxetic acid-enhanced MRI, T2*WI and SWI at the baseline visit; (**B**) the images of gadoxetic acid-enhanced MRI, T2*WI and SWI at the follow-up visit. After effective 3-month treatment, the patients received gadoxetic acid-enhanced MR scan again, and the images showed the abnormality was improved significantly. PVP: portal-venous phase; HBP: hepatobiliary phase.

Image feature of gadoxetic acid-enhanced, SWI and T2*WI were shown in Table 2. Firstly, image features of gadoxetic acid-enhanced MRI in hepatobiliary phase (HBP) were analyzed in the PAs-induced HSOS patients. Heterogeneity of liver parenchyma in HBP occurred in 100% of the PAs-induced HSOS patients. Heterogeneity of liver parenchyma on HBP appeared heterogeneous hypointensity which represents lower signal. Interobserver agreement between the two radiologists for evaluating the properties of hypointensity was excellent. Among these patients, distributional properties of heterogeneous hypointensity was diffuse in 9 patients (32.14%), multifocal distribution (mild) in 4 patients (14.29%), multifocal distribution (severe) in 15 cases (53.57%) (Fig. 1). Importantly, the severity of hypointensity occurred in HBP was precisely assessed by volume fraction of hypointensity (Fig. 2). The result showed that less than 25% of the total volume (mild) was involved in 13 cases (46.43%), 25–50% of the volume (moderate) in 13 cases (46.43%), and >50% of the volume (severe) in 2 cases (7.14%). Secondly, image signs of SWI and T2*WI were determined in the PAs-induced HSOS patients. Hypointensity in SWI and T2*WI was observed in the patients with PAs-induced HSOS(Figs 3 and 4). Thirdly, we measured the ratio of hypointense area to total area in 3 slices (Fig. S1), and then distributional similarity of heterogeneous hypointensity in different phases of MRI scan was investigated. Distributional similarity of hypointensity between portal-venous phase of MRI and SWI/T2*WI was observed in 71.43% of the patients; while, only 42.86% of the patients had similar hepatic distribution between hepatobillary phase and SWI/T2*WI. It indicated that the distribution of hypointense in SWI/T2*WI was similar to that of portal-venous phase (Figs 3 and 4).

**Table 2:** Image feature of gadoxetic acid-enhanced, susceptibility-weighted imaging and T2* imaging in the patients with PAs-induced HSOS.

Hepatobillary phase (Gadoxetic acid-enhanced MRI)	Patients with available data	Value
Hypointense	28	100% (28/28)
**Hypointense in hepatobillary phase**		
**Distribution**		
multifoca distribution (mild, <10 spots)	28	14.29% (4/28)
multifocal distribution (severe, ≥10 spots)	28	53.57% (15/28)
diffuse distribution	28	32.14% (9/28)
**Volume fraction of heterogenous hypointensity in hepatobillary phase**		
normal	28	0
<25% of the total volume (mild)	28	46.43% (13/28)
25–50% of the total volume (moderate)	28	46.43% (13/28)
≥50% of the total volume (severe)	28	7.14% (2/28)
T2* weighted imaging		
heterogenous hypointensity	14	100% (14/14)
**Susceptibility-weighted imaging (SWI)**		
heterogenous hypointensity	14	100% (14/14)

### Correlation of hypointensity on HBP with hypointensity in SWI/T2* imaging, liver function tests and the prognosis

Firstly, we determined the correlation of hypointensity in HBP of gadoxetic acid-enhanced MRI with that in different phases of MRI scan. The Pearson test demonstrated area ratio of hypointense in HBP was positively correlated in different phases of MRI scan (Table 3). Secondly, the correlation between the severity of heterogeneous hypointensity and a panel of liver function tests was determined. As shown in Table 3, the severity of hypointensity in HBP of gadoxetic acid-enhanced MR scan was positively correlated with the synthetic function of liver, which as revealed by prothrombin time (PT), INR. Finally, prognostic factors of the PAs-induced HSOS patients were determined using univariate logistic regression. The data of 27 consecutive patients were analyzed in our study because one patient without follow-up data was excluded. The median follow-up of the 27 patients was 2.3 years (range 1.4–4.3 years), 8 patients (29.63%) died. The result showed the severity of hypointensity in HBP was a risk factor of death events (OR = 25.500) in the patients with PAs-induced HSOS, while albumin (ALB) was a protective factor (OR = 0.661) (Table 4).

**Table 3:** The correlation test of hypointensity in SWI/T2*WI and liver functional test with the severity of hypointensity in HBP of gadoxetic acid-enhanced MRI.

Variable	Hypointensity in different phases of MRI scan^a^			Liver functional test^b^	
	Hypointensity in portal phase (the ratio of area, 3 sections)	hypointensity in SWI (the ratio of area, 3 sections)	hypointensity in T2* (the ratio of area, 3 sections)	PT	INR
	n = 28	n = 14	n = 14	n = 28	n = 28
Correlation coefficient	0.756	0.805	0.83	0.397	0.393
*P* value	<0.001	<0.001	0.002	0.036	0.038

Note: ^a^the ratio of hypointense area to total liver area in HBP of gadoxetic acid-enhanced MRI (3 slices) was used; ^b^volume fraction of hypointensity was used.

**Table 4:** Results of logistic regression analysis of prognostic factors. Univariate logistic regression was conducted to determine whether liver function tests and the severity of hypointensity in HBP predicted the prognosis of the PAs-induced HSOS patients.

Variable		Beta	S.E.	*P* value	OR	95% CI
A panel of liver function tests	ALT	−0.001	0.005	0.778	0.999	0.988~1.009
	AST	0.001	0.004	0.817	1.001	0.993~1.009
	Albumin	−0.414	0.174	0.017	0.661	0.470~0.928
The severity of hypointensity in HBP (<0.337)		reference				
The severity of hypointensity in HBP (≥0.337)		3.329	1.107	0.003	25.500	2.912~223.273

Note: one patients was excluded due to the lack of follow-up data.

## Discussion

Hepatic sinusoidal obstruction syndrome is a complication of hematopoietic stem cell transplantation (HSCT) and use of certain chemotherapeutic agents. In China, HSOS can be caused by ingestion of herbs containing pyrrolizidine alkaloids. In this study, we described the result of laboratory examination, imaging features of gadoxetic acid-enhanced and susceptibility-weighted MR imaging in PAs-induced HSOS. Firstly, abnormal liver function was observed in most of the PAs-induced HSOS patients. Secondly, we found heterogeneous hypointensity in HBP of gadoxetic acid-enhanced MR image occurred in 100% of the PAs-induced HSOS patients. Thirdly, hypointensity in SWI and T2*WI was observed in the patients with PAs-induced HSOS, and the distribution of hypointensity on SWI and T2*WI was consistent with heterogeneous hypointensity in portal-venous phase of MRI scan in most of the patients. Finally, the severity of heterogeneous hypointensity scored by volume fraction in HBP of gadoxetic acid-enhanced MRI was positively correlated with PT and INR, the severity of hypointensity in HBP was a risk factor of death events. Previous studies scored hypointensity/hypoattenuation according to area ratio or signal intensity^10,11,17,19^, and our study demonstrated the severity of hypointensity scored by area ratio or signal intensity was not correlated with coagulation function (PT and INR) (data not shown). It indicated that our strategy was more accurate than previous methods in defining the severity of hypointensity^9–11,17,19^.

One of pathological changes in PAs-induced HSOS is the extravasation of erythrocytes into the space of Disse in zone 3^7,8^. Infiltrated macrophages engulf erythrocytes containing hemoglobin to degrade it, producing hemosiderin. T2*WI is a gradient-echo sequence, and SWI is a gradient-echo technique that utilizes phase information. Both of them detect paramagnetic substances such as hemoglobin and hemosiderin. So, hypointensity in SWI and T2*WI was demonstrated in PAs-induced HSOS patients. Importantly, SWI and T2*WI displayed the lesions more clearly than any other pre-contrast sequences. In summary, SWI/T2*WI provide a useful adjunct to MRI in diagnosing PAs-induced HSOS, and are applied to the HSOS patients with severe renal insufficiency or contraindication to contrast materials in future.

It is well-known that dynamic contrast-enhanced MRI provides information about the perfusion in vascular phases. In HSOS, RBCs, leucocytes and cellular debris penetrate into the space of Disse, which results in the obstruction of sinusoidal flow. Then, the thickening of the subintimal zone leads to the narrowing of the venular lumen and an increased resistance to blood flow^7,20^. Thus, portal-venous phase of enahnced MRI scan showed the change in the perfusion resulted in heterogeneity of liver parenchyma^10^. Simultaneously, macrophages engulf erythrocytes to produce hemosiderin in lesion, which is demonstrated by hypointense in SWI and T2*WI. The extravasation of RBCs leads to decreased perfusion of liver parenchyma and the deposition of hemosiderin in the area of lesions, which demonstrates the distributional similarity of hypointensity in SWI and T2*WI and portal-venous phase of enhanced MRI scan. Since gadoxetic acid is taken up via an organic anion transport system by normal hepatocytes, hepatobiliary phase of gadoxetic acid-enhanced MRI reveals liver function. When massive hepatocytes necrosis occurs and the uptake of gadoxetic acid is damaged, thus hypointensity was observed in HBP of gadoxetic acid-enhanced MRI. Vascular phases of DCE MRI provide information about the perfusion, hepatobiliary phase (HBP) reveals the function of hepatocytes. Thus, these lead to the difference in the distribution of hypointensity between SWI and HBP of gadoxetic acid-enhanced MRI (Fig. 3). In addition, our study demonstrated the severity of hypointensity in HBP correlated with liver function (Table 3). As shown in Fig. 4, a 59-year-old male patients showed typical imaging features of MRI after the intake of PAs. After 3-month treatment, damaged liver function restored to normal and hypointensity on HBP of gadoxetic acid-enhanced MRI disappeared. Thus, HBP of gadoxetic acid-enhanced MRI revealed liver function in PAs-induced HSOS patients.

HSOS occurrs in the patients who receive cytoreductive therapy prior to hematopoietic stem cell transplantation(HSCT), oxaliplatin-based chemotherapy for metastatic colon cancer, or used herbal remedies containing PAs^7,8^. In the patients receiving oxaliplatin-based chemotherapy, reticular hypointensity was observed in HBP of gadoxetic acid-MRI, which was highly specific in detecting HSOS^17,18,21^. In HSOS patients (2 cases) after HSCT, MRI features consisted of hepatomegaly, hepatic vein narrowing, periportal cuffing, gallbladder wall thickening, ascites, pleural effusion and heterogeneity of liver parenchyma^22^. In the HSOS patient (1 case) who used a recreational drug during anal intercourse, hepatomegaly. heterogeneous hypointensity were observed in DCE MRI^23^. In summary, all these indicated that the radiologic signs of MRI were various in the patient of HSOS due to the different etiologies. Heterogeneous hypointensity was a common feature of MRI scan. However, the degree of hypointensity and involved area were different between oxaliplatin-induced HSOS and PAs-induced HSOS. Compared with oxaliplatin-induced HSOS, the involved areas and the degree of hypointensity are more obvious in the PAs-induced HSOS.

Obviously, our study had several limitations. Firstly, it was retrospective study, not prospective study. Selection bias occurred in retrospective cohort studies. Secondly, control patients were not included in our study. HSOS should be discriminated from Budd-Chiari syndrome (BCS) and congestive heart failure due to the similarities in clinical manifestations and liver histology among HSOS, BCS and congestive heart failure. Fortunately, BCS was excluded because the patency of the inferior vena cava and hepatic veins was demonstrated through imaging modality containing True FISP MRI sequence. Finally, liver biopsy was not performed in most of the PAs-induced patients due to thrombocytopenia, clotting abnormalities and extensive ascites; therefore, we could not correlate histological change in the liver with hypointensity of HBP.

In conclusion, heterogenous hypointensity of liver parenchyma on HBP of Gadoxetic acid-MRI and hypointensity in SWI and T2*WI was observed in the PAs-induced HSOS patients; thus, it will provide evidences for the diagnosis of PA-induced HSOS. In addition, the severity of hypointensity in HBP of Gadoxetic acid-MRI correlated with liver function. However, further study should be performed to confirm our results and investigate the mechanism underlying imaging features of gadoxetic acid-enhanced MR imaging.

## Materials and Methods

### Patients selection

**Figure 5:**
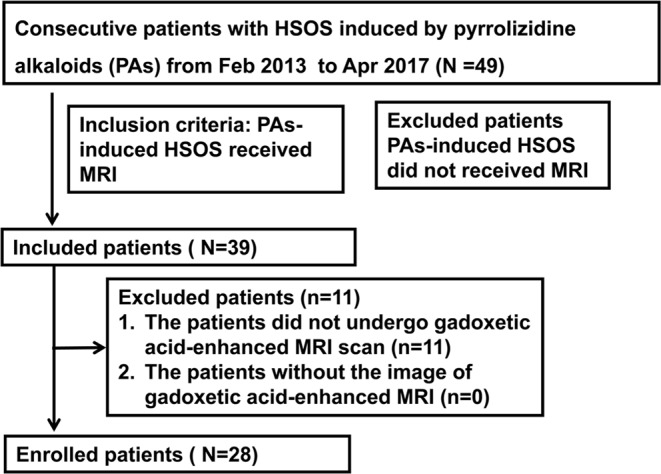
Flowchart of the patients selection, inclusion, and exclusion.

28 consecutive PAs-induced HSOS patients were enrolled in our study from Feb, 2013 to Apr, 2017, all the enrolled patients with HSOS were caused gynura segetum (*Tusanqi*) containing PAs (Fig. 5). Diagnostic criteria were (i) the patients met the modified Seattle criteria for HSOS characterized by hyperbilirubinemia, hepatomegaly and weight gain due to fluid accumulation; (ii) a Roussel Uclaf Causality Assessment Method (RUCAM) score ≥ 3; and (iii) a history of ingestion of gynura segetum (*Tusanqi*). The patients have chronic liver diseases, hepatocellular carcinoma, Budd–Chiari syndrome (BCS) or congestive heart disease were excluded^5,9–11^. Inclusion criteria for patient recruitment were as follows: (A) PAs-induced HSOS undergone gadoxetic acid-enhanced MRI scan. (B) the image data of gadoxetic acid-enhanced MR imaging were obtained. Exclusion criteria were: (A) HSOS patients caused by gynura segetum did not receive gadoxetic acid-enhanced MR; (B) the image data were not obtained from a picture archiving and communication systems (PACS) (Fig. 5).

### Data collection

Medical records of the recruited patients were retrospectively reviewed, and then the pertinent data of the patients extracted from medical records were tabulated in a database. The pertinent data of the patients included their age, gender, presenting symptoms and signs, medical history, laboratory results of blood test, imaging findings (X-ray, ultrasonography, CT, MRI, positron emission tomography/computed tomography and *et al*.), the results of ascitic fluid analysis, pathological changes (if available).

### MRI Technique

All MRI examinations were conducted with the 1.5 T MR-scanner (Magnetom Avanto, Siemens Medical Solutions, Erlangen, Germany), using a body phased array coil. The standard MR image sequences and the parameters were listed in Supplementary Table 1. T2*-weighted imaging (T2*WI) and susceptibility-weighted imaging (SWI) were obtained in 14 cases. The images of dynamic and HBP were acquired using T1-weighted gradient echo sequences with fat saturation. In brief, a dose of 0.025 mmol/kg gadoxetic acid (Primovist; Bayer, Leverkusen, Germany) was intravenously administered with a flow rate of 1 mL/s, and flushed with 20 mL saline. Imaging delay time was determined using a bolus tracking technique. Images were collected in arterial, portal-venous, equilibrium phases with multiple acquisitions between 20–180 s. Hepatobiliary phase images were acquired at 5, 10, 15, 20 min respectively after intravenous bolus injection of gadoxetic acid.

### Imaging Processing and Analysis

All MR images were independently reviewed by two experienced radiologists, who were unaware of clinical data, and interobserver agreement of the independent analysis was assessed using kappa (κ)statistics. Whereas discrepant diagnosis was jointly re-evaluated to reach a final consensus. Firstly, we assessed the distribution of heterogeneous hypointensity in hepatobillary phase (HBP). Since scatter distribution of heterogeneous hypointensity was observed within the liver parenchyma, hepatic distribution of hypointensity was classified: mild multifocal lesions (<10 spots), severe multifocal lesions(≥10 spots), diffuse lesions (Fig. 1). Secondly, we scored the degree of hypointensity according to signal intensity^11,17^ and found various degree of hypointensity in most of the patients; thus, we did not classify the degree of hypointensity through signal intensity. To assess the severity of heterogeneous hypointensity accurately, we measured the volume of hypointensity, and calculated volume fraction of hypointensity (Fig. 2). Volume fraction of hypointensity was calculated as followed: (1) the dynamic MRI image series were firstly performed with a bias correction (N4 Bias Correction Algorithms implemented in Simple ITK) to remove the B1 non-uniformity artifact, and then with a motion correction implemented in Elastix (http://elastix.isi.uu.nl) using pre-contrast phase as the refs^24,25^. (2) The hepatobillary phases were then linearly fitted in a pixelwise manner to generate the slope and offset volumes respectively. The unnormal regions were then identified as regions both appearing as hyperintensity in the slope volume and hypointensity in offset volume and were measured using the 2-class threshold segmentation algorithm implemented in Scipy (https://www.scipy.org). The volume fraction was then calculated as the ratio of unnormal region over the whole liver region which was manually drawn. 3) A 3-point ordinal scores were used to analyze the severity of heterogenous hypointensity: mild, less than 25% of the total volume involved; moderate, 25–50% of the area was involved; and severe, >50% was involved. Finally, we calculated the ratio of hypointense area to total liver area in 3 different slices (the level of right hepatic vein, left sagittal section of portal vein, portal vein trunk) (Fig. S1); and determined distributional similarity of the lesions in different phases of MRI scan.

### Statistical Analyses

Continuous variables are presented as means and standard deviation and categorical variables as numbers and percentage. The correlation between the severity of heterogeneous hypoattenuation and laboratory findings was determined by using the Pearson test. The cut-off value of the severity of hypointensity in HBP was determined by Youden index (Table 4). Univariate logistic regression was conducted to determine whether liver function tests and the severity of hypointensity in HBP could predict the prognosis. The interobserver agreement for assessment of imaging findings was determined using the κ-statistic. The level of agreement was defined as follows: poor, κ-values of 0.20 or less; fair, κ-values of 0.21–0.40; moderate, κ-values of 0.41–0.60; good, κ-values of 0.61–0.80; and very good, κ-values of 0.81–1.00. A *P* value less than 0.05 was considered indicative of a significant difference. Statistical analyses were performed using SPSS version 17.0.

### Ethics approval and consent to participate

The retrospective study was conducted in accordance with the Declaration of Helsinki and was approved by Ethics Committee of Tongji Medical College, Huazhong University of Science and Technology. The requirement for informed consent was waived because of retrospective study.

## Supplementary information

Data.1

## Data Availability

The data in this study are available from the corresponding author on reasonable request.
