# Supplementary material for: Gadoxetic Acid-Enhanced Hepatobiliary-Phase Magnetic Resonance Imaging for Pyrrolizidine Alkaloid-Induced Hepatic Sinusoidal Obstruction Syndrome and Association with Liver Function
Source: Sci Rep. 2019 Feb 4;9:1231. doi: 10.1038/s41598-018-37775-1 (PMC6362127; doi:10.1038/s41598-018-37775-1)
Supplement: Supplementary file 1 — Data.1 [file 41598_2018_37775_MOESM1_ESM.pdf]

Supplementary Data

Gadoxetic Acid-Enhanced Hepatobiliary-Phase Magnetic Resonance Imaging for Pyrrolizidine Alkaloid-Induced Hepatic Sinusoidal Obstruction Syndrome and Association with Liver Function

Tingting Guo[1], Xin Li[1], Xiaoqian Yang[2], Xiangquan Kong[1], Hui Liu[3],

Tao Bai[2], Keshu Xu[2], Jin Ye[2], Yuhu Song[2]

[1] Department of Radiology, Union Hospital, Tongji Medical College, Huazhong University of Science and Technology, Wuhan 430022, China.

[2] Division of Gastroenterology, Union Hospital, Tongji Medical College, Huazhong University of Science and Technology, Wuhan 430022, China;

[3] 12Sigma Technologies, Shanghai 200000, China

Material and methods:

Distributional similarity of heterogeneous hypointensity in different phases of MRI

To investigate distributional similarity of the lesions in different phases of MRI scan, the ratio of hypointense area to total liver area was calculated. In brief, the region of hyperintensity (green line, Fig S1) and total liver (yellow line, Fig S1) were manually drawn in 3 different slides (the level of right hepatic vein, left sagittal section of portal vein, portal vein trunk), and then the total liver area subtracted hyperintensity area was hypointense area. The percentage of hypointense area to total liver area was calculated. The mean calculated from three slides was used for the evaluation.

Supplementary table 1MR Imaging Parameters for All Sequences Used in the Study in 1.5T MR system

Sequenceand Imaging Plane	Repetition Time (msec)	EchoTime (msec)	Section Thickness(mm)	Flip angle	Field of View (mm)	Matrix size	Bandwidth (Hz/pixel)
T2W-HASTE,coronal	1500	90	6/1.2	170	400	256*256	425
T2W TSE with fat saturation,axial	2000	80	6/1.2	150	380	207*384	260
T1W GRE(flash 2D) in phase and opposed phase, axial	160	2.38/4.76	6/1.2	70	380	192*256	390
Echo-planar imaging turbo SE DW imaging, axial	1800	70	6/1.2	none	380	144*192	1736
SW imaging, axial	40	20	5/0.8	15	380	168*256	380
T2* imaging, axial	200	11.9	6/1.2	20	380	184*256	390
T1W 3D GRE(vibe) SPAIR, axial	4.74	2.38	3/0	10	380	250*320	400
T1W 3D GRE(vibe) Q-fat saturation, coronal	2.97	1.1	2.5/0	10	400	207*288	600
True FISP, axial	3.86	1.93	5/1	61	380	220*256	400

Note. DW=diffusion weighted, FLASH=fast low-angle shot, HASTE=half-Fourier acquisition single-shot turbo spin echo, SE=spin echo, SPAIR=spatial adiabatic inversion recovery, T1W=T1 weighted, T2W=T2 weighted, 3D=three-dimensional, 2D=two-dimensional, VIBE=volumetric interpolated breath-hold examination, SWI=susceptibility-weighted Imaging

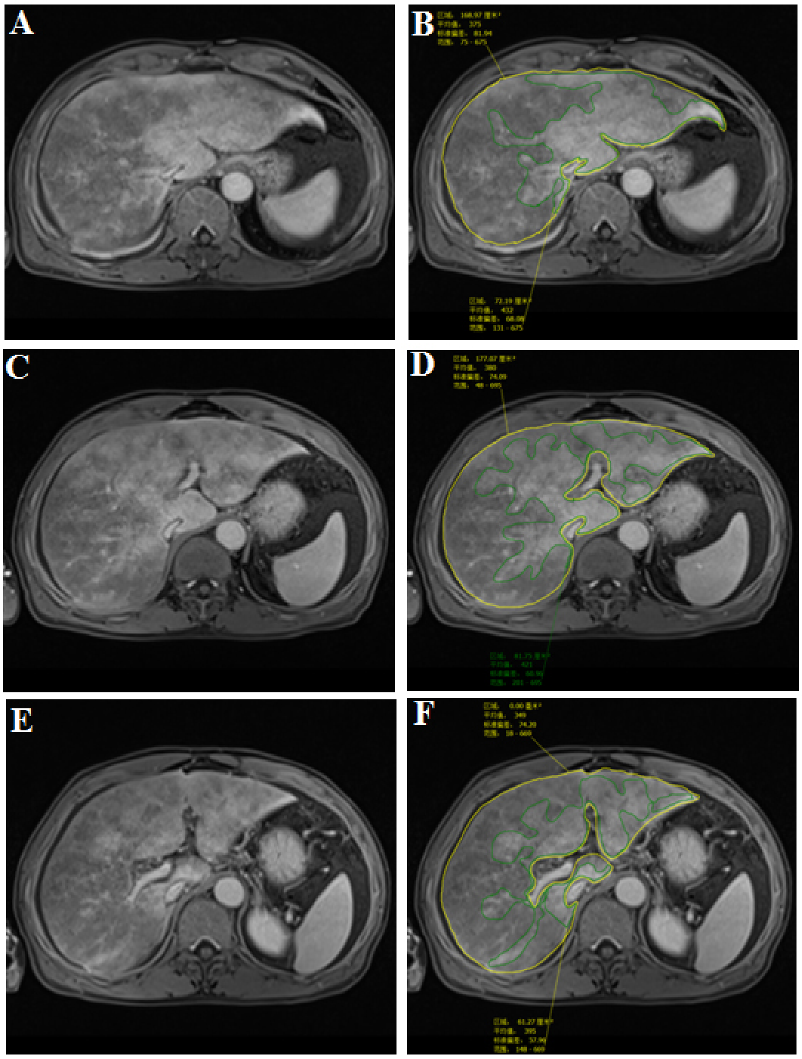

Figure S1. Schematic diagrams of the strategy for calculating the ratio of hypointense area to total liver area in portal-venous phase of DCE scan. The ratio of hypointense area to total liver area= [total liver (yellow line)-hyperintensity (green line)]/total liver [yellow line]. A, B: right hepatic vein level on portal-venous phase of DCE MRI scan; A: MR imaging, B: outline of hyperintensity (green line) and total liver (yellow line) in MR imaging; C, D: left sagittal section of portal vein level; and E, F: portal vein trunk level.
